# What has single‐cell RNA sequencing revealed about microglial neuroimmunology?

**DOI:** 10.1002/iid3.362

**Published:** 2020-10-21

**Authors:** Norwin Kubick, Patrick C. Henckell Flournoy, Pavel Klimovich, Gina Manda, Michel‐Edwar Mickael

**Affiliations:** ^1^ Department of Biochemistry and Molecular Cell Biology (IBMZ) University Medical Center Hamburg‐Eppendorf Hamburg Germany; ^2^ Department of Immunology PM Research Center Stockholm Ekerö Sweden; ^3^ Department of Radiology Victor Babes National Institute of Pathology Bucharest Romania; ^4^ Department of Pathology Bevill Biomedical Sciences Birmingham Alabama USA; ^5^ Department of Experimental Genomics, Neuroimmunology Group, Institute of Genetics and Animal Biotechnology Polish Academy of Science Magdalenka Poland

**Keywords:** microglia, single‐cell RNA sequencing, T cells

## Abstract

The use of single‐cell RNA sequencing (scRNA‐seq) in microglial research is increasing rapidly. The basic workflow of this approach consists of isolating single cells, followed by sequencing. scRNA‐seq is capable of examining microglial heterogeneity on a cellular level. However, the results gained from applying this technique suffer from discrepancies due to differences between applied methods characteristics such as the number of cells sequenced and the depth of sequencing. This review aims to shed more light on the recent developments that happened in this field and how they are related to the methods used. To do that, we track the progress and limitations of various scRNA‐seq methods currently available. The review then summarizes the current knowledge gained using scRNA‐seq in the field of microglia, including novel subpopulations associated with function and development under homeostasis as well during several pathological conditions such as Alzheimer, lipopolysaccharide response, and HIV in relation to the methods employed. Our review points out that despite major developments found using this technique, current scRNA‐seq methods suffer from high cost, low yields, and nonstandardization of generated data. Additional development of scRNA‐seq methods will raise our awareness of microglia's heterogeneity and plasticity under healthy and pathological conditions.

## Graphical abstract

Single‐cell RNA sequencing (scRNA‐seq) reveals that microglia vary by age, brain regions, and by disease. However, scRNA‐seq is still facing challenges the biggest of which is sequencing costs

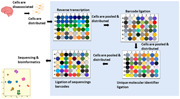

## 1 INTRODUCTION

### 1.1 Role of single‐cell RNA sequencing (scRNA‐seq) deciphering the role of microglia

**Figure 1:**
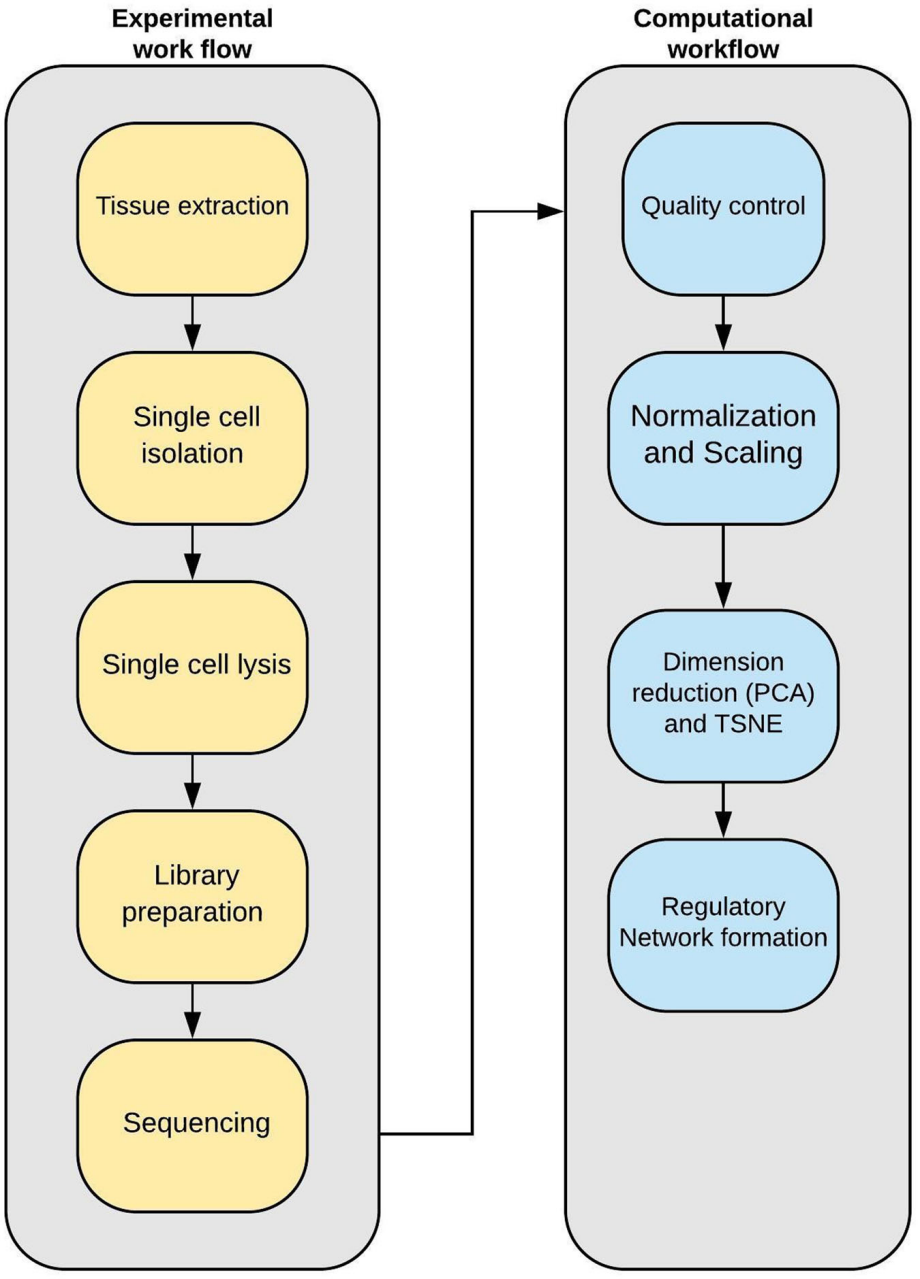
Experimental and computational workflow for single‐cell RNA sequencing. Different techniques could be used for isolating single cells. This stage includes isolating single cells and unique barcoding of transcriptomes

scRNA‐seq have shed more light on microglial interactions on the level of individual cells.^1^ The workflow for this approach compromises the isolation of single cells, followed by sequencing (Figure 1).^2^ This simple yet innovative technology has revolutionized the field of microglial neuroimmunology. Applying scRNA‐seq has unraveled several unique cell types in the brain, encompassing adaptive, innate immune cells as well as neurons.^3^ It was also used to reveal unique subpopulations such as proliferative‐region‐associated microglia (PAM), among others, that could be involved in disease prognosis.^4^, ^5^, ^6^, ^7^, ^8^ The main objectives of this technique are to investigate cellular distribution in the brain^9^ as well as to track cellular development.^10^, ^11^ The future of this method is promising, with more innovation expected to reduce the cost and reveal complex gene regulatory interactions.^12^ In this review, we summarize some of the advancements in microglial immunology made through scRNA‐seq. The review is divided into three sections. Section 1.1 will briefly summarize the main building blocks of the scRNA‐seq (i.e., single‐cell isolation and computational and analysis). Section 1.2 will cover some of the critical findings that were achieved by scRNA‐seq, highlighting opportunities for further research. Finally, in Section 1.3, we will discuss alternative approaches for scRNA‐seq.

#### 1.1.1 Single‐cell isolation techniques

**Figure 2:**
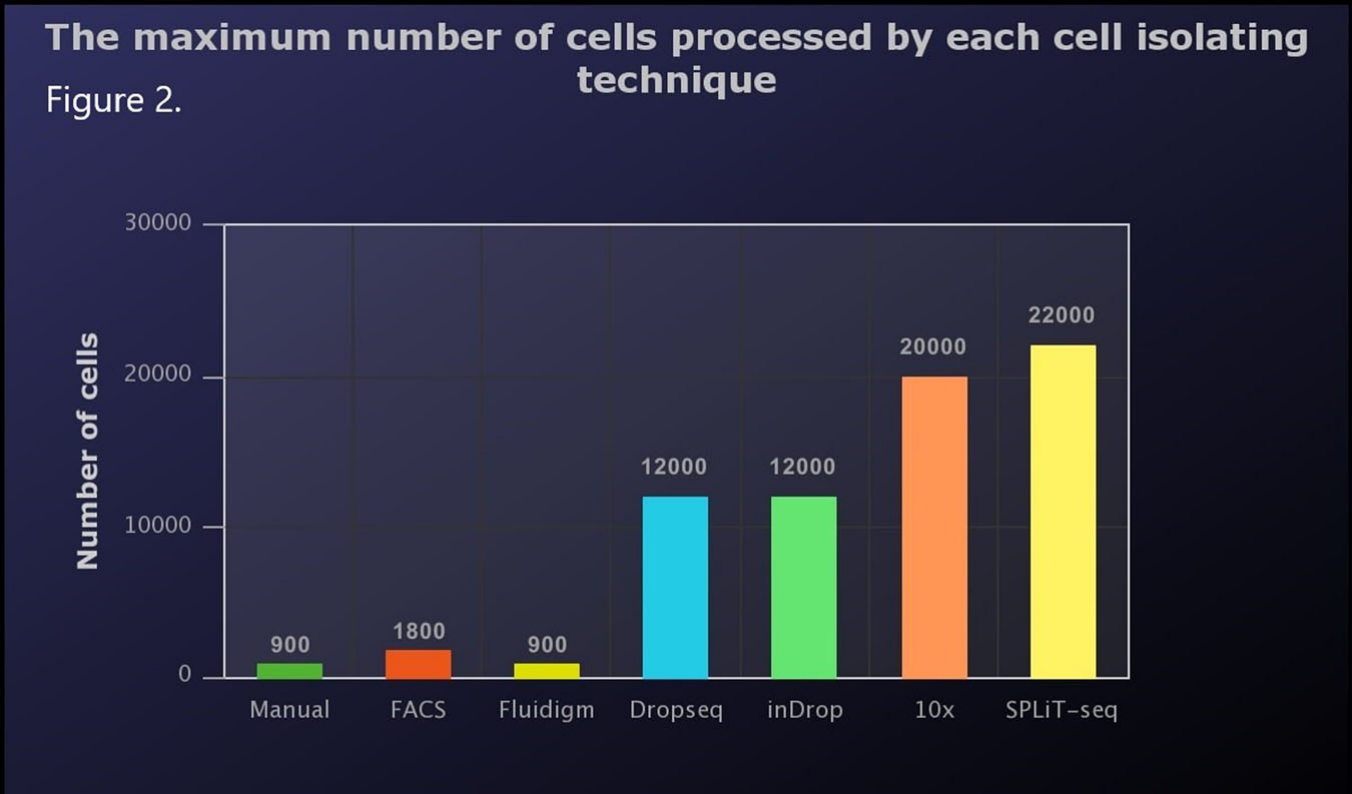
Comparison of different attributes of single‐cell RNA sequencing techniques. (A) The maximum number of cells processed by each cell isolating technique. SPLiT‐seq can process up till tens of thousands of cells per run, whereas Fluidigm can process only 800 cells per run. Using fluorescence‐activated cell sorting (FACS) implies having a presumption on the subpopulation. Dropseq, inDrop and 10x are all drop‐based systems, however, the number of cells acquired by 10x is larger than the two other methods. This is also reflected in the price of opertaion

**Figure 3:**
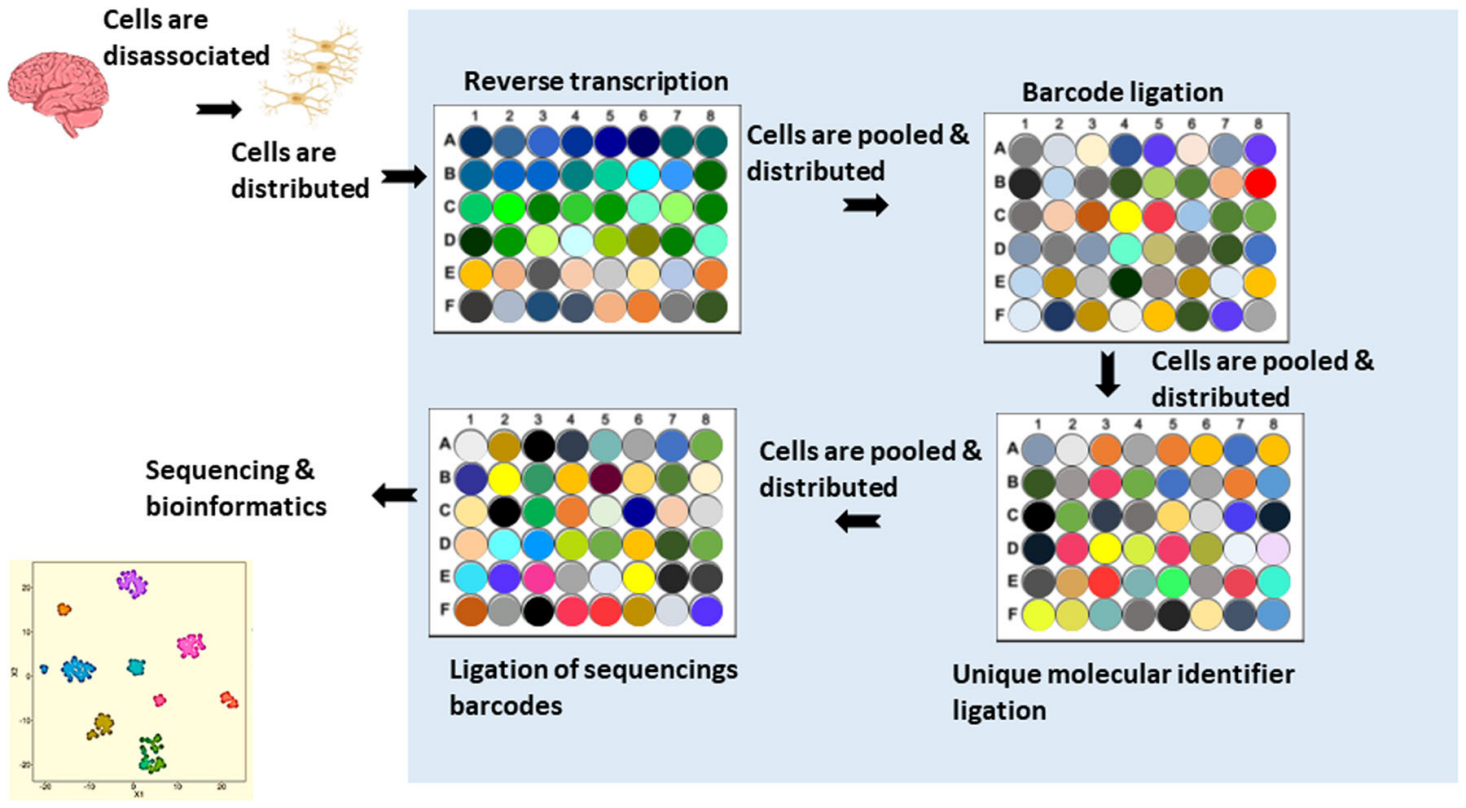
Workflow of SPLiT‐seq single‐cell approach. After disassociating the cells, they pass through four stages of distribution and pooling using a unique well identifier in 96‐well plates. In the first stage, reverse transcription with well‐specific primers takes place. Second, the ligation of the unique well barcode is performed. Third, ligation of a different unique molecular identifier per well is done. Finally, ligation of sequencing barcodes is completed

Various methods could be used to achieve a single cell suspension. Manual picking continues to be utilized to isolate single cells for RNA sequencing. This method has been used to explore microglial presence in the cortex area of the embryo.^9^, ^10^ Typically, this technique is performed after collagenase and papain digestion of the central nervous system (CNS). Single cells are then randomly picked into individual tubes. The main advantages of this method are accuracy and a low number of doublets. However, it is time and power‐consuming. Fluorescence‐activated cell sorting (FACS) augmented with single‐cell RNA‐seq has been extensively used in microglial studies. Using this combined approach, researchers were able to identify novel microglial subpopulations^5^, ^8^ and studied the effect of pathogens.^13^, ^14^ Following tissue homogenization, the cells of interest are selected and sorted using FACS into 384‐well plates.^14^ The main disadvantage of this method is that it is framed by already known assumptions about cell clustering. Automatic cell capture and isolation started with Fluidigm integrated fluidic circuit. C1 system vigilantly isolates single cells into separate reaction chambers. Fluidigm C1 workflow could be employed in a broad range of applications, including whole transcriptome analysis, targeted gene expression profiling, and gene regulation.^15^ However, the maximal capacity of the system is limited. Newer versions of this system can analyze up to 800 cells/run, which is still lower than other approaches described in Table 1 and Figure 2. Droplets based techniques have been used to explore the neuroimmune response to pathogens^13^ as well as neuroinflammation^16^ on a cellular level. Typically, droplet‐based techniques use a microfluidic device, where each cell is lysed and barcoded separately followed by sequencing (e.g., using Illumina HiSeq‐4000 sequencer). These techniques include Dropseq,^17^ 10x,^18^ SeqWell,^19^ and inDrop.^20^ Dropseq was employed to investigate the effect of lipopolysaccharide (LPS) in microglia, revealing two distinctive subpopulations that are different in their major histocompatibility and complement profiles.^13^ SeqWell was utilized to examine the effect of HIV on the cerebrospinal fluid (CSF),^7^ detecting several subpopulations of microglia.^7^ Also, 10x in conjunction with FACS was applied to investigate microglial heterogeneity in the aging brain, and revealed nine different novel subpopulations. It is important to note that these droplets techniques differ considerably in their capacity. For example, Drop‐seq can process over 10,000 cells. However, it suffers from low cell capturing efficiency with a cost of $0.2 per cell.^21^ Conversely, 10x can achieve higher call capturing efficiency but with a higher cost of $1.2 per cell^22^ (Table 1 and Figure 2). Split‐pool ligation‐based transcriptome sequencing (SPLiT‐seq) has excellent potential to further explain immune interactions with the CNS (Figure 3). This approach produces one of the highest yields in scRNA‐seq.^23^ The cost for library preparation using this technique is also one of the lowest ($0.01/cell) (Table 1). Using this technique Rosenberg et al.^23^ produced 150,000 single‐nucleus transcriptomes from young mice CNS clustered into various and distinct populations in both the brain and the spinal cord. The reason behind the above‐mentioned high yield is that instead of using physical partitioning systems, the workflow treats cells as individual compartments. Split‐seq utilizes the mathematical principle of combination to generate a large number of unique barcodes (Figure 3). This method is based on using multiple stages of spreading the cells in well plates and pooling them again. In the first stage, cells are spread among various wells of a 96‐well plate, where each cell is tagged with a primer specific to its respective well, and reverse transcription is performed and the cells are pooled together. Second, a redistribution step takes place, where a well distinctive barcode is appended to each cDNA. In the third step, a unique molecular identifier is ligated in a new 96‐well plate. This step is repeated once more into another 96‐well plate. A user might opt for a 384‐well plate to increase the yield further. One of the downsides of this technique is the probability of failing to identify genes with low expression.^23^ However, the authors did not focus on the immune cells in the brain and did not produce a complete analysis of reported microglia.

**Table 1:** Comparison between various types of scRNA‐seq suitable for neuroimmunology experiments

Technique	Cost/experiment ($)	Cost/cell ($)	Maximum number of cells/run	Cell capturing efficiency (%)	Mean reads/cell
Manual picking + Smartseq2	3600 sequencing + consumables	?	900	80	100,000
FACS + Smartseq2	3600 sequencing FACS running costs	11	1800	Low capture rate for rare cells	100,000
FACS + CEL‐seq2	3600 sequencing + consumables	?	1500	Low capture rate for rare cells	?
Fluidigm	2000 consumables + 3600 sequencing	17	~800	41	486,026 (aligned)
Dropseq	3600 sequencing	0.25	>10,000	Low (10?)	20,000–40,000
inDrop	3600 sequencing + consumables	7	10,000	~45	20,000–40,000
10x	2000 consumables + 3600 sequencing	1.2	20,000	46% (Wang et al. 2019)	207,046 (prealignment)
SPLiT‐seq	3600 sequencing + 900 consumables	0.01	22,000	30–50	40,000

Abbreviations: FACS, fluorescence‐activated cell sorting; scRNA‐seq, single‐cell RNA sequencing.

#### 1.1.2 Data analysis workflow

**Figure 4:**
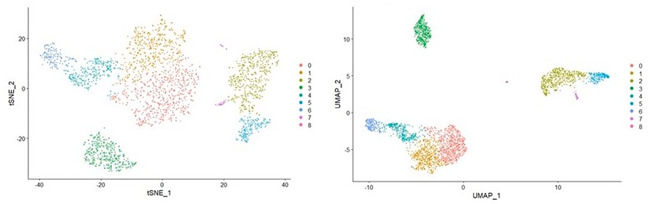
Comparison between the t‐distributed stochastic neighbor embedding (t‐SNE) and Uniform Manifold Approximation and Projection (UMAP) dimensional reduction techniques. The PBMC3K data set was used to compare the abilities of t‐SNE and UMAP. The PBMC (peripheral blood mononuclear cells) data set was produced utilizing a 10x workflow that employed PBMC isolated from a healthy donor and sequenced using NextSeq 500. It should be noted that t‐SNE and UMAP produce similar results in smaller datasets. However, the similarity is decreased in larger datasets

Microglial scRNA‐seq data analysis normally utilizes the following workflow: (a) Quality control, (b) Data normalization and scaling, (c) dimension reduction and visualization, and (d) interactome analysis. Quality control (QC) is designed to choose the datasets which are valid for further interpretation.^23^, ^24^ One of QC's parameters is the number of the aligned reads per cell, where cells with an exceptionally low or high number of reads are omitted. In the context of neuroimmunology, it is critical to consider the biological variation between cell types investigated (e.g., microglia vs. T cells, activated vs. naive, migrant vs. resident) as this might influence the final data accuracy. Large numbers of mitochondrial transcription are commonly used as indicators of cell stress, and therefore, cells with elevated mitochondrial gene expression are often not included in the analysis. After performing the quality control check, the normalization phase takes place, which is important to eliminate batch effects. Due to the scarcity of data and the possibility of inability to capture the messenger RNA (mRNA), scaling of sequencing data is performed to equalize the expression levels of selected host genes in all cells. The third step is size reduction and visualization. PCA is commonly used to estimate cell relativity based on differential gene expression.^21^ PCA can reduce the dimensions of the data to a limited number of components, where each component represents a trend in the variation of gene expressions among the cells. This technique allows the user to cluster the cells according to variation among the cells. However, in the case of large data, PCA suffer from overcrowding issues in the case of scRNA‐seq. To tackle this problem other techniques such as the t‐distributed stochastic neighbor embedding (t‐SNE) and Uniform Manifold Approximation and Projection (UMAP).^22^ t‐SNE is efficient in the representation of microglial heterogeneous subpopulations as clusters. However, t‐SNE is not able to deduce intercluster relationships. This technique is also time‐consuming. UMAP^22^ is a newly published technology aimed at preserving local and global data structures with a short runtime (Figure 4).^25^, ^26^ One of the critical revelations that scRNA‐seq can uncover about microglia cells status is their trajectory in health and disease. Trajectory inference is predominantly used to investigate cellular progression through dynamic processes. The main hypothesis behind this technique is that cells during a certain pathological or developmental process are transitioning from one state to another. The input to this analysis is the PCA, along with the gene list of the top differentially expressed genes. Normally, the data dimensions are first reduced further. The result is then used to build a pseudo‐time graph. This time‐series graph assumes that cells that cluster together, based on their genes expression are in the same state and that the cells that cluster nearest to them are their trajectory. On the basis of this information, the graph can take several shapes such as linear, tree, and circular. Several R packages could be used to build time‐series graphs such as Moncole,^27^ Slingshot,^28^ and TSCAN.^29^ One of the main limitations of this process is the need for prior knowledge about the expression of the genes, to increase the biological relevance of the data. One way to improve this limitation is to build time‐series graphs using integrated scRNA‐seq samples isolated from the same subject at different time points as this does not require prior knowledge of the biological status of the samples. The final step of the analysis would be to investigate the interactome. One way to achieve this is to implement GOAE (Gene Ontology Autoencoder) or GONN (Gene Ontology Neural Network), where genetic interaction networks are created using neural networks.^30^ These two methods can be combined with GO further to understand the biological meaning behind the formed networks.

#### 1.1.3 Out of the box pipelines

Multiple box outlines can be used to analyze data generated by single‐cell RNA‐seq.^31^ scPipe summarizes data quality in a report that could be saved in HTML format.^31^ It also produces the count matrix used in downstream analysis, including normalization, visualization, and statistical testing.^31^ Different R packages such as Scater and Seurat^32^ could be used to analyze scRNA‐seq data. Seurat allows users to investigate microglial heterogeneity generated using the 10x approach utilizing specific functions that take three inputs: Expression matrix, barcodes, and genes (features). The output is a PCA in the form of a t‐SNE plot.^33^ The user can also use customized markers to identify different microglial clusters based on their expression. One of the interesting questions that face the field of scRNA‐seq is identifying cellular types from the resulting t‐SNE clusters. The general assumption that if a certain cellular maker (e.g., AQP4, a known marker of astrocytes) is highly expressed in a certain cluster, it is assumed that this cluster represents that specific cell type (e.g., astrocytes). This assumption, although logic, neglects the effect of up and downregulation in pathological conditions. One of the downsides of using Seurat is the extensive computer power needed to analyze large datasets. Moana is a hierarchical machine‐learning framework that can classify data from different datasets.^34^ For example, this framework could be used with larger datasets to construct tissue‐specific cell type atlases. With the production of large amounts of scRNA‐seq data, the integration of different cell groups arising from different studies is an important task. Seurat 3.0 allows data integration.^33^ It is worth noting that the Seurat R library contains methods for merging datasets to a common context and transferring information from the query data set context. Another alternative is scQuery, which is a web server integration tool.^35^ The webserver takes in the RPKM matrix as an input, and the output is the t‐SNE plot.

**Figure 5:**
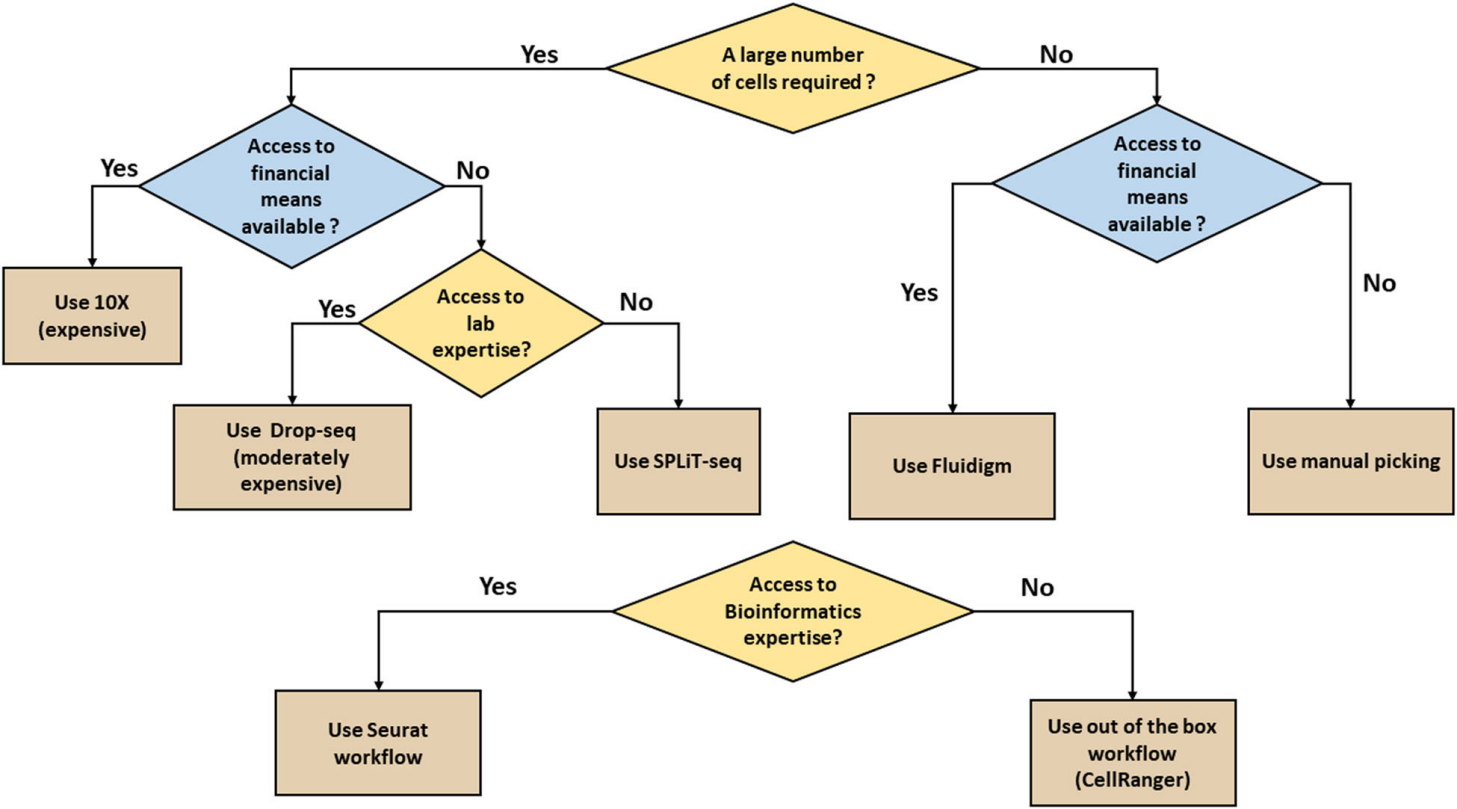
How to choose the best scRNA‐seq to suit your project needs. The main issue that defines which workflow to choose is the required number of cells. A higher than 1000 cells requirement can be achieved using Droplets methods or through Split‐seq. If the laboratory have access to bioinformatics expertise, it may be better to build a custom bioinformatics workflow using Seurat

The field of bioinformatics techniques for the analysis of scRNA‐seq data is quickly evolving. We would like to refer the reader to more focused reviews such as.^21^, ^24^, ^36^, ^37^ The analysis of the available scRNA‐seq methods shows that the right choice of the method depends on several factors, including resources available, the required number of cells, and the availability of bioinformatics expertise (Figure 5).

The following section of the review will focus on the unresolved questions of the microglia role in the immune system. Then we will focus on the efforts that have been made to investigate these issues using single‐cell RNA sequencing and will emphasize the unanswered questions regarding the role of the peripheral immune cells in the CNS. Finally, we will discuss the results obtained by various research groups studying these cells using scRNA‐seq and will explore available alternatives techniques.

### 1.2 Microglial findings based on scRNA‐seq and open questions

#### 1.2.1 Investigation of microglia location and development within the embryonic brain using scRNA‐seq approaches

The phenotypic and functional characterization of microglia remains challenging.^38^ Cell lineage, subpopulations, and frequency of microglia are still a matter for debate^39^ Microglia develop from erythro‐myeloid progenitor cells located in the yolk sac in a similar fashion to tissue‐resident macrophages. Embryonic microglia develop through the interactions of various transcription factors including RUNX, CEBP, and IRF, then under the influence of IL34 and CSF1R, microglia can reach maturation.^40^ In the past, microglia were classified into the phenotypes M1 (proinflammatory) and M2 (anti‐inflammatory). However, both M1 and M2 have variable molecular signatures and may include various subpopulations.^41^ For example, transforming growth factor‐b (TGFb) seems to be critical for microglia function.^42^ Paradoxically, deleting TGFb seemed to result in directing microglia into an inflammatory macrophage phenotype.^43^ Moreover, it was found that a microglial signature gene “Sall1” maintained microglial identity in vivo.^43^ A recently found microglial subpopulation is called disease‐associated microglia (DAM) phenotype in neurodegenerative disease, chronic neuroinflammatory states, and, in addition, aging.^44^ However, whether these various clusters of microglial cells constitute distinct subpopulations is still not clear. If microglia subpopulations can be clustered based on their TGFb receptor expression is also an exciting question.

Single‐cell sequencing can answer critical questions about microglia origin during human embryonic development.^45^, ^46^, ^47^ It could also reveal whether microglial distribution differs in frequency between brain compartments^4^ and is exceptionally suitable to resolve the open question of investigating microglia subpopulations' heterogeneity. Microglia development was found to be unsynchronized in different brain regions.^9^ Healthy humans' embryonic cerebral cortex region was investigated using manual picking RNA single‐seq.^9^ The results identified 4000 single cells from various brain regions during mid‐gestation in embryos.^9^ There was a significant difference in microglia numbers and shapes between different brain regions. For example, the medulla had a large number of microglia that presented an amoeboid phenotype. Conversely, the IG regions had a lower number of microglia, and they appeared to have a ramified form. The study also revealed three microglial CD68 + subclusters (Table 2).^9^ However, no functional difference or specific makers were shown to differentiate between them. A similar study used manual picking to examine microglia development in the prefrontal cortex (PFC) in embryos.^10^ This study demonstrated that microglia progenitors develop from the peripheral mesodermal tissue. They also showed that microglia appeared early in PFC and were residents during the PFC development. The early appearance and stable population of microglia in the developing PFC, especially in the intermediate zone, support a model in which microglia regulates neuronal apoptosis, neurogenesis, and synaptic pruning in development (Table 2).^9^ It was also possible to reveal that microglia migrate from the outside ventricular zone during the 8 weeks to the subventricular zone and the intermediate zone during the 12 and 16 weeks, respectively. Finally, microglia infiltrate the ventricular zone and the cortical plate around the 19th and 23rd weeks, respectively. Another study employed FACS followed by Smart‐seq2 to investigate microglia development during three main stages: (i) Embryonic (ii) postnatal, and (iii) adult mice. Remarkably, the authors found that microglia heterogeneity is inversely proportional to age, with older mice having less heterogeneous microglia populations. The reason behind that is unclear. Would that mean that humans lose microglia heterogeneity as they age? Would these subpopulations play a role in defending against neuroinflammation? One of these populations that could be lost to age is PAM and is mainly found in mice developing white matter. However, if this subpopulation exists in humans is still unknown. There is a resemblance between PAM and DAM. Would that mean that microglia reprogramming takes place based on the environment, or do both populations exist side by side in older humans? Finding out the difference between these populations could prove critical in fighting neuroinflammation diseases where DAM's role is not sufficient.^4^

**Table 2:** Immune cells new features identified by scRNA‐seq

Tissue	Animal	Status	Age	Cell type	Observation
Cerebral cortex	Human	Healthy	Embryonic	Microglia	Medulla microglia is more in number but less developed than the IG region
					Identified three microglial sub‐clusters
PFC	Human	Healthy	Embryonic	Microglia	Tracked microglia appearance in the PFC, ventricular zone and SVZ
CSF	Human	HIV	33–59 years	Microglia	Novel myeloid subset in CSF with gene expression characteristics of neurodegenerative disease‐associated microglia
Brain	Mice	FNX	8‐weeks old	Microglia	Identified a consensus microglial common to several neurodegenerative diseases
Brain	Mice	AD and ALS	Adult mice	Microglia	DAM subpopulation that is Trem2‐dependent
Brain	Mice and Humans	Healthy mice, in five different clinical stages of EAE and in end‐stage HD	E14.5–P530	Microglia	Major finding in that study was a novel subset of microglia that selectively express the chemokine CCL4
Brain	Mice	LPS fed	3–4 months	Microglia	A classical activated proinflammatory microglial profile accompanied by a decreased homeostatic signature
Hippocampal nich	Mice		6 and 10 weeks	Microglia	In young mice, dentate gyrus microglia expressed high levels of Csf1r, Cx3cr1, and Tyrobp
					Detected a small number of Cd45high+ cells‐Dentate gyrus microglia could be have been primed toward an M2
					Dentate gyrus microglia could be have been primed toward an M2
					During aging, the relative proportion of microglia seems to increase, but their molecular profile remains unchanged

Abbreviations: AD, Alzheimer's disease; ALS, amyotrophic lateral sclerosis; CSF, cerebrospinal fluid; DAM, disease‐associated microglia; EAE, the experimental autoimmune encephalomyelitis; FNX, facial nerve axotomy; HD, Huntington's disease; LPS, lipopolysaccharide; PFC, prefrontal cortex; SVZ, subventricular zone.

#### 1.2.2 scRNA‐seq reveals more about microglia role in aging

The role of microglia in aging was investigated using scRNA‐seq in several reports.^48^ One report examined the difference in microglial phenotype using a time point approach, including during development and in old mice. The study also investigated the change in the microglia population in the case of brain injury (Table 2). After purifying the microglia using FACS, cells were sequenced utilizing Illumina NextSeq 500 sequencers with a depth of 500,000 reads. The authors were able to identify various distinct transcriptionally different microglial populations. One of these populations selectively expressed the chemokine CCL4.^48^ The numbers of this unique population followed a positive trajectory, where it increased during aging and injury. It seems that this population can have a pathogenic effect as it expressed several proinflammatory chemokines, including CCL3, 7, 9, and 12. Besides, this population was capable of producing proinflammatory cytokines such as IL1b as well as TNF‐α, suggesting an ability to recruit other proinflammatory immune cells. In another report,* Artegiani et al*.^49^ investigated the microglial phenotype in the hippocampal dentate gyrus during aging through sorting cells using FACS and then applying a variation of Cel‐seq2. Their data revealed that in that particular area known for its function in pattern recognition, learning, and memory, a population that is marked by upregulated expression of Csf1r, Cx3cr1 as well as Tyrobp exists. This study suggests that in the aging dentate gyrus, microglia could have an M2‐like phenotype. However, the authors did not present any behavioral studies that pinpoint this population's role in pattern recognition and learning. It will be interesting to perform a functional analysis to investigate how these subpopulations affect memory processing during aging. Another important aspect that is still poorly understood is the difference in population frequency among different brain regions during aging. These questions can be readily answered using scRNA‐seq.

#### 1.2.3 scRNA‐seq reveals more about microglia role in Alzheimer's disease (AD)

Microglial heterogeneity in AD was recently investigated.^8^ In that report, the authors employed scRNA‐seq using FACS, followed by MARS‐seq in Tg‐AD mice. This study identified a unique microglial subpopulation and called it “disease‐associated microglia” (DAM). What is special about DAM is their capability of taking up Aβ particles. The authors proposed that DAM function through two phases: (i) Initiation of activation and (ii) activation of the TREM2 pathway. First, to start the activation process, MafB, which plays a regulatory function in lineage specificity, is downregulated along with other microglial checkpoints. Second, a TREM2‐dependent pathway is activated where phagocytic, and lipid metabolism activity is enhanced. The reason behind the inability of this unique microglia subpopulation to stop AD progression is still unknown. Interestingly, further analysis also identified DAM in amyotrophic lateral sclerosis (ALS) models (Table 2).^8^ Another study that also employed scRNA‐sequencing to study microglia in AD was performed by Tay et al.^5^ This study utilized FACS, followed by CEL‐Seq2 on Fluidigm chips to sequence the transcriptome of 1536 single cells. Their findings pointed toward a neurodegeneration‐associated transcriptome similar to DAM (Table 2).^5^ However, there was variation in several genes, including FOS, Jund, KLf7, and Sgk1. KLF7 belongs to a family of regulators of transcription called, Kruppel‐like factors. FOS and Jund is a component in the AP‐1 pathway, also known to regulate the transcription of genes. Sgk1 is also a Serine/threonine‐protein kinase that is known to play a role in cell proliferation. These observations indicate that there could be a difference between DAM and the microglial population identified by Tay et al.^5^ It is not yet clear why these populations are different if they are fighting the same disease. Other unresolved questions about the nature of DAM include their point of appearance with respect to disease progression. Also, their origin is also not known. These reports indicate that there are various unsolved questions regarding microglia in AD.^50^

#### 1.2.4 LPS effect on microglia

scRNA‐seq revealed a unique microglial phenotype that appears under LPS injection.^13^ LPS is a known endotoxin that induces an acute response in the body.^51^ Thus it is safe to assume that there will be a shift of microglia transcriptomic profile into proinflammation in response to LPS injection. However, the exact phenotype of this population was not yet known.^13^ Using FACS, followed by Dropseq, allowed the authors to discover that proinflammatory genes such as *IL1b, Tnf*, and *Ccl2* were upregulated in LPS mice compared to steady‐state. Conversely, Mef2C, which is known to regulate the microglial inflammatory response, was downregulated, along with CD206 (Mrc1), which supports a neuroprotective phenotype.^52^, ^53^ Intriguingly, the main difference between this LPS specific microglia and DAM is the downregulation of phagocytosis genes (Tyrobp and Trem2). It has been widely thought that phagocytosis is correlated with anti‐inflammatory response.^54^ However, it has been shown that phagocytosis of myelin increased proinflammatory signals. Thus, further research is still needed to clarify this point.

#### 1.2.5 Investigating microglia heterogenity in CSF during HIV

Farhadian et al.^7^ studied the subpopulations controlling the immune response associated with HIV infection (Table 2). Here, the authors used scRNA‐seq employing SeqWell to phenotype the immune cells in the CSF from blood samples of HIV‐infected individuals with virus‐induced suppression. The results revealed that 5% of the cells investigated resemble DAM and exhibit ist gene expression characteristics. As expected, the DAM have a high expression of TREM2 and APOE, AXL, and TREM2. Compared with other myeloid subsets identified, this subpopulation expressed higher levels of CTSB, APOC1, and MSR1 (CD204), which are also known to play a major role in neurodegeneration diseases.^7^ DAM activation during HIV infection is likely to be caused by the ability of HIV to cause neurodegeneration. How DAM help fight HIV is an intriguing question that still needed to be answered.

#### 1.2.6 Can scRNA‐seq lighten up the road to better understand the interaction between peripheral cell‐mediated immunity and microglia?

Exploiting the scRNA‐seq ability to understand the interaction between adaptive immunity cells and microglia is almost nonexistent. Immune cells' ability to access the brain without requiring local trauma was previously demonstrated.^55^, ^56^ T lymphocytes were shown to be present in normal human cerebrospinal fluid.^57^ However, the interaction between these migrating cells and the CNS, including (i) their point of entry, functional analysis for (ii) supporting neurogenesis, and (iii) memory formation is far from complete. Surprisingly, the location of peripheral adaptive immune cells to the brain is still controversial. Three locations have been proposed (i) the arteries of the choroid plexus, (ii) the perivascular space meningeal blood vessels, and (iii) postcapillary venules.^58^ A simple scRNAseq experiment in the experimental autoimmune encephalomyelitis (EAE) mice might solve this dilemma. During brain development, the peripheral immune system performs a vital role in neurogenesis, gliogenesis, and synapse formation.^59^ It was indicated that B1a cells were abundant in the neonatal mouse brain.^59^ Depletion of B1a cells during brain development resulted in reducing oligodendrocyte‐precursor cells (OPCs) numbers.^55^ By neutralizing the soluble receptor Fcα/μR secreted by B1a cells, OPC proliferation was inhibited, and the proportion of myelinated axons in neonatal mouse brains was reduced.^55^ It would be crucial to investigate the difference in B1a distribution between the different brain regions using scRNA‐seq in connection to microglial distribution. scRNA‐seq can also detect the change of the trajectory of the B1a population during healthy aging. It could also uncover new B‐cell subpopulations that could be interacting with microglia during neurogenesis development. Moreover, there is evidence that adaptive immune cells could be implicated in learning and memory.^60^, ^61^ Mice deficient in Rag1 and Rag2 (which are responsible for the diversity of T and B cells) display impairment in various cognitive tests and suffer from distorted neurogenesis.^61^ T lymphocytes were also shown to be essential for the maintenance of hippocampal neurogenesis.^62^ Additionally, it was reported that CNS‐specific T cells could influence cell regeneration and plasticity in the hippocampus. Activated Th2‐like cells infiltrate the meninges during learning tasks and produce anti‐inflammatory, neuroprotective cytokines (e.g., IL‐4 and IL‐10), and hence enhance cognitive functions. Severe combined immunodeficiency (SCID) mice manifest cognitive deficits and behavioral abnormalities. Interestingly, impaired cognition in SCID could be treatable by T cell restoration.^63^ An important question arises about the interaction between microglia and adaptive immune cells under homeostasis. Can specific microglia subpopulations contribute to the selective infiltration of specific immune cells to the brain under homeostasis? Single‐cell analysis can reveal more about the relationship between microglia and the controversial adaptive immune cells' role under neuroinflammation. The depletion of Tregs increases the speed of decline of cognitive abilities in APPPS1 mice. Besides, IL‐2 treatment, which is known to increase Treg proliferation, selectively increased the numbers of plaque‐associated microglia and improved cognitive functions in APPPS1 mice. It is unknown if this interaction was direct through the upregulation of IL2R receptors on the microglial surface or through another indirect mechanism. Adoptive transfer of Tregs in 3xTg‐AD improves the prognosis of Alzheimer's in 3xTg‐AD mice. This was mirrored in improvement in cognitive abilities and reduction of the Aβ amyloid. Paradoxically depleting Treg in another model of Alzheimer known as 5xFAD lead to converse results, manifested by improving cognitive abilities. Why is there a difference in Treg function between the two Alzheimer models? Treg can have various subpopulations such as RORγt Treg and IL17+ FOXP3+ Tregs that can differ considerably from classic Treg. Could the difference in the effect lie in the heterogeneity of Tregs infiltrating the brain between the two models? Could it be that microglia are influencing one group positively, whereas negatively affecting the others? Further scRNA‐seq can enhance our understandings of these intriguing questions? Proinflammatory CD4+ T cells such as Th1 and Th17 were shown to release proinflammatory cytokines that can increase inflammation during AD. However, little is known about direct interactions between Th17 and resident microglia. Even more, increased numbers of CD8+ T cells was reported in the brain AD patients. However, how do CD8+ T cells contribute to AD is not fully understood, and if CD8 infiltration to the brain during AD is affected by microglia is also not known. scRNA‐seq might be able to answer many of these intriguing questions. One attempt to employ scRNA‐seq to study EAE mouth models was described by Gaublomme et al.^64^ The authors applied a scRNA‐seq approach using Fluidigm C1 with 976 Th17 cells^1^ to study immune cells function during EAE. The results predicted that Th17 cells have a spectrum of subpopulations, with two extremes, the first having a regulatory function, while the other extreme subpopulation can induce pathological effects.^60^ The study revealed new regulators associated with these opposing states such as *Gpr65, Plzp, Toso*, and *Cd5l*.^64^

#### 1.2.7 Limitations of scRNA‐seq experiments

Irrespective of their high innovation, single‐cell sequencing techniques still suffer significant flaws. For instance, it is challenging to pinpoint a sequence depth that is appropriate for any given experiment. Overall, most scRNA‐seq techniques only produce a few thousands of genes compared to multiple thousands of genes provided by microarray experiments. Another flaw is the need for standardization of the identified clusters. For example, different microglia scRNA‐seq studies identified several distinct subclusters,^5^, ^7^, ^8^ which may have the same expression patterns. Computational approaches that could compare and build interstudies correlations to identify the same subpopulations across the same type of cells are urgently needed. However, the variation in the number of cells between experiments could constitute a challenge to building such correlations.

### 1.3 Available alternatives

There are several alternatives for scRNA‐seq. These alternatives could also be used to investigate microglial interactions using the single‐cell approach. These methods include single‐cell nuclei isolation, single‐cell mass spectrometry (scMass‐spec), single‐cell DNA sequencing, and single‐cell ATAC‐seq (scATAC‐seq).

#### 1.3.1 Single‐cell nuclei isolation

Single‐cell nuclei isolation is generally compatible with both frozen tissue and droplet techniques. Hence, it is suitable for processing brain‐bank‐derived tissues. Isolating single nuclei is based on targeting nuclear mRNA. Investigating the difference in transcriptomic profile between cellular and nuclear mRNA revealed a high degree of similarity between the two entities. Also, there is high concurrency between bulk mRNA genes enriched in the nucleus and that of single nuclei RNA. This technique allowed researchers to sequence more than 15,000 nuclei in an investigation that targeted the spinal cord in mice and particularly in the lumbar area. The research revealed the existence of microglia in that region. Interestingly, the research did not investigate further microglial subpopulations. Furthermore, the nature of the microglia found, its phenotype, and resemblance with DAM or LPS microglia have not been investigated. Further research is required to address these captivating inquiries.

#### 1.3.2 Single‐cell Mass spec

A powerful alternative to scRNA‐seq is the sc‐Mass spec. This technique allows exploring microglial heterogeneity on the protein level. In conjunction with FACS, this method was used to identify novel populations across multiple neurodegenerative diseases models, including Alzheimer's, EAE, and normal aging. This approach uses combinations of palladium isotopes to barcode unique cells. This technique's results were impressive, showing that there is a high degree of heterogeneity of immune cells even under homeostasis.^65^ This study was also able to separate microglia from border associated macrophages through the expression of CD38 and MHC2. Importantly the authors showed that microglia experience a change of its transcriptomic profile according to the pathological environment it is experiencing. Paradoxically, this approach could not detect DAMs but detected a distinct microglia subpopulation with upregulated levels of CD14. The reason behind the inability of this method to detect DAMs is still not clear. Interestingly they identified the same microglial subpopulation in aging mice but not in EAE, as EAE microglia has upregulation of MHC2 as well as Sca‐1. Another major difference between the two disease models that the entire microglial population became reactivated during EAE in contrast with AD, where only a subset of microglia showed a reactivation phenotype. This interesting research opens the door to other questions, such as why is there is a difference between different disease models? Could that be because of microglia plasticity? Is there a difference in the protein‐based description of heterogeneity and that of a transcriptome based? Can scRNA‐seq and sc‐Mass spec be done jointly or mapped together using the same mouse models?

#### 1.3.3 Single‐cell DNA sequencing

Single‐cell DNA sequencing is a promising single‐cell approach that could help to understand the role of microglia in cancer. It has been shown that in glioma, cancer cells enslave microglia to promote cancer growth rather than fight it. Why certain cancer cells have this ability while other cancer cells perhaps in the same tumor lack it? Is it because of the heterogeneity of mutations between cancer cells or the heterogeneity of microglia fighting them? One way to tackle this question is to use single‐cell DNA. Similar to scRNA‐seq, this approach is based on barcoding genomic DNA from cells using droplets techniques.^66^ The barcodes are then employed to differentiate between different sampled cells. This method was shown to be successful in investigating heterogeneity within acute myeloid leukemia tumor populations.^66^ However, applying single‐cell DNA sequencing in microglia biology is still in its infancy.

#### 1.3.4 Single‐cell ATAC‐seq

scATAC‐seq is an integrated platform that consists of two main parts: (i) ATAC‐seq, and (ii) microfluidics platform.^67^ The ATAC‐seq is an assay that allows transposases to access open chromatin and then sequencing it. Transposes attaches to DNA in regions of open chromatin. The more open the chromatin, the more transposase will attach and cut the DNA. One downfall of this technique is the contamination of mitochondrial DNA. It is important to use computational methods to discard mitochondrial DNA contamination that could reach up to 34% of the total sequenced DNA. The integration of ATAC‐seq with a programmable microfluidics platform (Fluidigm) allows performing ATAC‐seq using individual cells. The main goal of scATAC‐seq is to determine if there is a change in chromatin accessibility between various cells or at different time points during disease progression.

#### 1.3.5 CITE‐seq versus REAP‐seq

Integrating transcriptome and proteome profiles may reveal hidden aspects of microglial interactions. Currently, two techniques, namely, CITE‐seq (cellular indexing of transcriptomes and epitopes by sequencing), and REAP‐seq (RNA expression and protein sequencing assay) are capable of determining protein and transcriptome levels in single cells. In^68^ the authors showed that using CITE‐seq improved defining the degree of heterogeneity among natural killer cells. Moreover, REAP‐seq^69^ helped the researchers discover a new CD8 T‐cell population that is CD34, CD38, CD123, CD117, CD13, CD33, and HLA‐DR positive. However, how it differs from conventional CD8 cells is not yet known. The two mentioned techniques employ a similar approach, where proteins are detected by using antibodies conjugated to a DNA sequence. These antibodies‐derived tags are encapsulated in droplets together with unique cells and microbeads as in the case of conventional droplets‐based scRNA‐seq. However, the two methods differ in how the DNA barcode is conjugated to the antibody. Antibodies used in CITE‐seq are conjugated to streptavidin without being covalently bound to biotinylated DNA barcodes. REAP‐seq, on the other side, depends on covalent bonds between the antibody and aminated DNA barcode. However, both techniques have yet to be applied in the field of microglial neuroimmunology.

## 2 CONCLUSIONS

scRNA‐seq has revolutionized our appreciation of microglial heterogeneity; however, further progress is required. The most striking findings revealed using scRNA‐seq is that microglia in the CNS form a spectrum, with distinctive subpopulations lying on the extreme. For example, the DAM has an upregulated phagocytic activity, and on the contrary, LPS‐specific microglia have an attenuated phagocytic response.

Nevertheless, further experiments are needed to validate the difference between these various subpopulations. The instruments and methods employed to perform cell isolation, barcoding, and sequencing need to be cost‐effective (Table 1). SPLiT‐seq can produce billions of cells profiles in a single experiment.^23^ However, sequencing these profiles would cost more than one million dollars. Hence, a reduction in the cost of sequencing will help further push the limits of scRNA‐seq. The other obstacle facing scRNA‐seq is integrating the data produced. Applying R libraries such as Seurat is intuitive and easy to handle; however, for larger datasets, the resources needed to analyze could be unavailable, especially for smaller labs without cloud services access. Advancement in handling large datasets on the sequencing level costs and the computational resources cost will amplify our knowledge of microglia heterogeneity and their interaction with other cell types. Captivating developments could include integrating more than one sequencing method to simultaneously measure several parameters (e.g., genome, transcriptome, methylome. It could also highlight the change of microglia population‐based on investigating microglia markers evolution and the effect of drugs^51^, ^66^, ^70^). An interesting topic also is the detection of different isoforms and alternative splicing cases of microglial markers.^71^ The effect of drugs on the Interaction between microglia and CD4+ T cells infiltrating the brain during neurodegenerative diseases is another one.^72^ These developments could support scRNA‐seq becoming a plausible alternative to classical cellular clustering methods.

## Data Availability

The scRNA‐seq was obtained from 10x genomics. The dataset data set is known as PBMC3K and it consists of 2700 cells PBMCs isolated from a person. The sample was sequenced using ILLumina NextSeq 500 and with around 70,000 reads per cell. The PBMC3k data used in this review was downloaded and is publicly available on https://support.10xgenomics.com/single-cell-gene-expression/datasets/1.1.0/pbmc3k?
